# Hydrostatic Pressure as a Sensing and Control Parameter for Fission-Nuclear Process †

**DOI:** 10.3390/s26092602

**Published:** 2026-04-23

**Authors:** Siya Lozanova, Avgust Ivanov, Chavdar Roumenin

**Affiliations:** 1 Center of Competence “Quasar”, 1113 Sofia, Bulgaria; lozanovasi@abv.bg; 2 Institute of Robotics, Bulgarian Academy of Sciences, “Acad. G. Bonchev” Str. Bl. 2, 1113 Sofia, Bulgaria; avgust.ivanov@abv.bg

**Keywords:** nuclear energy, hydrostatic pressure, radioactive matter, chain reaction, nuclear instrumentation

## Abstract

**Highlights:**

**What are the main findings?**

- Possibility for generation and control of the chain reaction with hydrostatic pressure is proposed;
- As pressure grows, the probability for a more substantial amount of neutrons from nuclei strongly increases;
- An instrumented titanium high-pressure chamber was designed, built and validated up to approximately 2 × 10^5^ atm using a weakly compressible inert surrogate fluid.

**What are the implications of the main findings?**

- The chamber provides the experimental platform required to test a proposed sensor effect in which hydrostatic pressure acts as an impact control for fission-material systems;
- Reduction in critical mass in chain reaction and diminution of nuclear fuel.

**Abstract:**

This study proposes a novel physical effect arising in radioactive matter: initiation and control of a nuclear chain reaction through high hydrostatic pressure. We present the design of a compression-assisted reactor consisting of a titanium chamber with a cylindrical channel, which can be filled with Deuterium in which Uranium _92_U^235^ clusters are dissolved. External energy is introduced gradually via a hydraulic piston, which considerably simplifies the reactor mechanics. As hydrostatic pressure increases, the effective interatomic distance decreases due to the overlap of inner electron shells, significantly raising the probability that neutrons released from fissile nuclei will collide with neighboring atoms rather than escape the medium. The safety mechanism is intrinsic to the design: when pressure is reduced, the reactor shuts down autonomously without external intervention. The technical feasibility of the chamber was validated using a weakly compressible inert fluid mixture of kerosene and transformer oil, confirming that the required pressure regime of 200,000 atm is mechanically achievable. The principal anticipated advantage of this effect is the possibility for reduction in the critical mass required to sustain a chain reaction. It corresponds with diminution in the quantity of nuclear fuel needed. Future experiments with radioactive materials could be conducted to develop the proposed phenomenon.

## 1. Introduction

The production of nuclear energy is due to the discovery of O. Hahn and F. Strassman in 1938. The root cause of this effect is that each neutron captured by the Uranium nucleus leads to its fission with the release of new neutrons and the generation of a too substantial amount of thermal energy. The process multiplies, resulting in an unfading chain reaction. For example, in a block of pure uranium _92_U^235^ or plutonium _94_Pu^239^, each neutron captured by the nucleus causes vibrations, spontaneous fission, whereas, after 10^−2^ s, the nucleus splits into two approximately equal parts (fission products). As a result, 2 or 3 neutrons are released, i.e., an average of 2.5 new neutrons. If the Uranium is in a less amount, which means a small mass, resulting in small volume and surface area of the structure, the main part of the generated neutrons leave the matter surface. After the neutrons come out of the product, the nuclear process tends to stop. However, if the Uranium mass exceeds a certain value G_0_ and volume, a very small part of the neutrons manage to leave it. This fixed amount of G_0_, known as critical mass, provides for the fission of new nuclei, more neutrons are released, etc., and the chain reaction occurs. Therefore, a minimum amount G_0_ of uranium _92_U^235^ is needed so that the chain process of the spontaneous fission of the nuclei does not subside. As is well known, if this process is not controlled, within a very short time, it encompasses a large number of nuclei and leads to a nuclear explosion accompanied by the release of enormous energy.

Nuclear systems containing enriched uranium are of paramount importance for the energy industry, in which the generation and management of a controllable, non-fading reaction of the fission process is perfectly mastered. For this purpose, special cylindrical cartridges with Uranium dioxide pressed into them are used. In about 1/3 of these cartridges, movable rods of the element Boron (B), regulating the fission process, are inserted. This element absorbs some of the so-called slow or thermal neutrons and thus the speed of the chain reaction is controlled, preventing a nuclear explosion. By cooling with water flowing under pressure of about 160 atm, the released thermal energy is absorbed and removed from the reactor in the form of steam. Such systems are used as primary sources of electrical energy. These installations are of considerable size, requiring appropriate geological, hydrological and especially, seismic conditions. The time period from the survey of the terrain, design, construction and international legalization of such systems requires up to 20 years. That is why, a characteristic trend in the design of nuclear reactors is the reduction in their size and shortening of the commissioning period [1,2,3,4,5,6,7,8,9,10,11].

Small modular devices for nuclear power generation are also known. Generally, they contain a cylindrical steel reactor. In its active zone, there are thin-walled tubular structures of identical size, made of stainless and heat-resistant steel. Each of these tubes contains another pair of tubes with fixed diameters—outer and inner, located radially and at a distance from each other. The cavity between each pair of these tubes is hermetically sealed and Uranium _92_U^235^ or Plutonium _94_Pu^239^ is pressed into it. The total amount of radioactive substance in the reactor exceeds slightly the critical mass G_0_. The pairs of tubes are located in a graphite or Deuterium medium, which controllably slows down the neutrons spontaneously released in Uranium. All the tubes in the reactor form an internal loop located within a surrounding case with a circulating cooling water flow. The heat from the chain reaction is removed from the reactor through a heat exchanger into an external steam loop, which activates a turbine that produces electricity [5,12,13,14].

Among the limitations of these modular reactors is the complicated design, due to the identical pairs of steel tubes and the radioactive substance hermetically pressed inside therein. An important requirement is the precise placement of the same amount of radioactive material in each cavity of the pairs of tubes. In emergency situations, it is imperative to safely interrupt the nuclear reaction. This is achieved by emergency removal of the Uranium tubes from the reactor or lowering into the reactor zone of other cylindrical graphite bodies, slowing down the process. However, this procedure requires a certain amount of time, which is sometimes insufficient.

In this article, a new concept has been presented for a sensor effect and a reactor based on it for generation and control of nuclear energy with high hydrostatic pressure is presented. Its applicability is planned in modular nuclear energy devices, simplifying the design and increasing their reliability. This research is the initial phase of a qualitative study into a new physical phenomenon. Future work will involve conducting experimental observations and incorporating them into an appropriate model of the complex processes occurring in the chain reaction under high hydrostatic pressure. The results obtained will be described in subsequent papers.

## 2. Materials and Methods

### 2.1. Operating Principle

The fission of Uranium is carried out by neutrons with relatively low energy, about 1 eV. These slow or thermal neutrons are energetically comparable to the chaotic thermal motion of molecules in gases. This process for the Uranium isotope _92_U^235^ can be written as follows:

(1) n01+U23592→B + C + N01 n,

where $n_{0}^{1}$ is the neutron causing the fission, and B and C are the fission products—isotopes of chemical elements from the middle of the Mendeleev table (barium, krypton, xenon, strontium, etc.), and N is the number of neutrons after the fission, N = 2, 3, 4.

These lighter nuclei have about 1 MeV higher specific binding energy than the heavy parent nucleus of _92_U^235^. This means that the nucleons in them are more tightly bound and have a lower rest mass. Part of the rest energy of uranium is converted into kinetic energy of the fission product. The process of obtaining nuclear energy includes the following stages. The physical state is characterized by the neutron multiplication factor K, defined by the expression P = (K − 1)/K. The following conditions are characteristic of the possible regimes of the nuclear process: (a) if K(t) > 1—the chain reaction increases with time t and the reactor is in a supercritical state where at P > 0, this is a state leading to nuclear explosion; (b) if K(t) < 1—the reactor fades away which means that it is below the critical state and is at rest, P < 0; and (c) if K(t) ≈ 1, P ≈ 0—the number of nuclear fissions is constant with time t and the reactor is in stable critical state, this being the so-called “golden” state for nuclear energy production. The criticality condition of the nuclear device is in accordance with the formula K = K_0_A = 1, where A is the part of the neutrons formed in the reactor that are absorbed in the active zone, i.e., in the chamber, and K_0_ is the neutron multiplication coefficient. It has been established that, under the action of neutrons, the nuclei of Uranium 235 or Plutonium 239 split into two parts, each one including half of their mass and 0.5 of their electrical charge [14].

The chain reaction proceeds after the following algorithm. The nucleus of Uranium captures one neutron; the impact of the neutron into the nucleus is accompanied by its vibration at increasing amplitude; then, the nucleus splits into two almost equal parts, releasing two or three neutrons. This reaction proceeds with significant release of thermal energy. The most essential part of this multiplication process is the fact that the neutrons released during fission cause the fission of other new nuclei. This splitting of atoms proceeds in geometric progression [1,2,3]. As a result, a non-fading chain reaction takes place—once it has started, it continues by itself. Uranium 235 or Plutonium 239 are preferred in nuclear energy because, with them, fission takes place under the action of fast and slow neutrons with arbitrary energy. It has been proven that it is better if the intrinsic energy of the neutrons is lower, thus they affect the nuclei more effectively. Moreover, the fission reaction does not fade away provided that the mass of the radioactive material is large enough. If necessary, graphite particles can be dissolved in Deuterium for more effective control of the nuclear process.

### 2.2. Sensor Effect Through Hydrostatic Pressure in Nuclear Processes

For the first time in nuclear physics and technology, a new physical effect arising in radioactive matter consisting of a generation and control of the chain reaction with high hydrostatic pressure is described [6,15]. This phenomenon brings the atoms closer together and the electrostatic bonds of the substance are broken while preserving the electric charge. The process leads to restructuring of the atomic configurations, including those of Uranium or Plutonium. The forces of the metallic bond are, by their nature, largely isotropic. The packing density of the atoms in the crystal lattice is directly related to the volume *V* of the crystal structure itself. The hydrostatic and monoaxial pressure **F** leads to an increase in the degree of ionization of the atoms. At high values of quantity **F**, an overlap of the inner electron shells is formed. In this case, the role of both the convergence of the atoms by pressure and their attraction by Van der Waals forces is important. The parameter *a*_Met_ of the crystal lattice in metals, i.e., the distance between the atoms, which are most often at the vertices of the lattice, decreases. Sharing of the electrons between the atoms takes place. The characteristic oscillatory motions of the ions are the reason why the crystal structure of metals, for example, Uranium, at any given moment of time is not strictly periodic. At non-zero temperature *T* > 0, the atoms in the lattice participate in thermal oscillations, whose energy E*_T_* is proportional to the temperature E*_T_* ~ *T*. However, the atoms also have a purely quantum exchange motion. These oscillations are a consequence of the Heisenberg uncertainty relation for the coordinate x and momentum p of the nucleus: ΔxΔp ≥ *ħ*, where *ħ* is Planck’s constant. The energy E*ħ* of these oscillations is also available at absolute temperature *T* = 0 [16]. In their synergy, these vibrational processes result in increased probability of a growing number of collisions in the regions between atoms, in which the neutrons leaving the nuclei fall. At the same time, the effective distance between the atoms *a*_Met_(**F**) decreases. Thus, conditions are created for the neutrons not to leave the surface of the substance _92_U^235^, including Uranium structures of smaller mass and volume, in which, without pressure **F**, the chain reaction would cease. Therefore, the high hydrostatic pressure **F** simultaneously strongly densifies Deuterium and brings the clusters and atoms of the radioactive material dispersed in it closer together. The described mechanism affects the amount of neutrons leaving the atoms, increasing their absorption by other nuclei, including when the ratio G < G_0_ is in effect. In the described process, for the first time, there is a prerequisite for the chain reaction to be controlled by hydrostatic pressure. Therefore, with the increase in the pressure **F**, more neutrons participate in the chain reaction. By regulating the pressure, the “golden” state of the reactor is also achieved: K ≈ 1, P ≈ 0. The number of nuclear fissions is practically constant with time t and the nuclear reaction is in a stable critical state. If the densification of deuterium with the radioactive material dispersed in it continues, a state most likely occurs in which K > 1:

(2) n01+U23592(F)→B + C + N[ξ(1/l(F)]01 n,

where l*, (l* ~ *a*_Met_), is the effective distance between Uranium clusters/atoms, ξ[(1/l*(**F**)] is the probability of the chain reaction occurring, the other notations are the same as in Equation (1).

The chain process increases with time *t* and the probability ξ that the reactor will fall into a dangerous supercritical state is also high, P > 0. However, if the pressure **F** is immediately removed, the state K < 1 is achieved quickly enough, the reactor fades away and is out of critical mode, P < 0. Deuterium D_2_O is used to slow down the avalanche effect in _92_U^235^ or plutonium _94_Pu^239^. An essential feature of the described sensor effect is that, with the help of the hydrostatic pressure **F**, it is possible to achieve conditions for the chain reaction to occur even with smaller amounts of uranium G than its critical mass G < G_0_(F = 0) [17,18,19,20,21,22,23].

## 3. Chamber Design for Implementation of the Physical Effect

**Figure 1:**
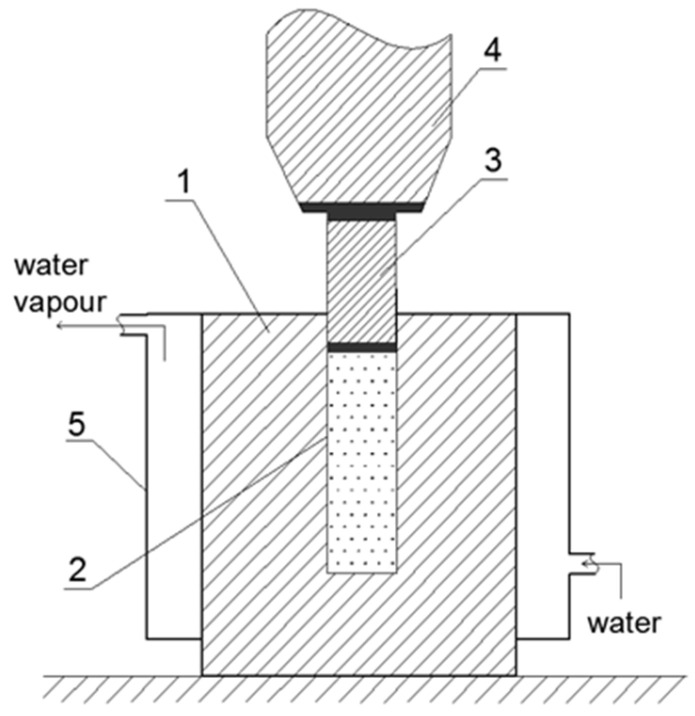
Schematic view of the nuclear system: 1—chamber; 2—channel containing Deuterium with dispersed uranium _92_U^235^; 3—piston; 4—press; 5—case with circulating water. The supercritical state of the nuclear reaction is neutralized by switching off the press 4 and relieving the pressure **F**.

The technology for implementation of the new physical effect is shown in Figure 1. The modular configuration contains a reactor with a neutron reflector, which should be filled with Deuterium (heavy water D_2_O) and clusters/atoms of Uranium _92_U^235^ or Plutonium _94_Pu^239^ dissolved in it. The reactor in turn includes a cylindrical thick-walled chamber 1 for high hydrostatic pressure with a capacity of up to several hundred thousand atmospheres and a cylindrical channel 2 along its length. It does not reach the bottom of chamber 1. The ratio of the external diameter d_ext_ to that of the chamber d_int_ is d_ext_/d_int_ ≥ 4.50.

There is also a cylindrical piston 3 with the diameter d_int_ of channel 2. It is subject to deformation monoaxial pressure exerted by press 4. In our case, the monoaxial pressure of press 4 in chamber 1 is transformed into hydrostatic pressure by Deuterium with _92_U^235^ small particles dispersed in it. The material from which chamber 1 for high hydrostatic pressure and piston 3 are made is titanium steel, which after certain procedures for increasing the hardness has a coefficient of about 150 Rockwell. By polishing and corresponding coating, channel 2 is a neutron reflector. The reactor is placed in case 5, surrounding the reactor, with water flow circulating in it at a pressure of about 160 atm, turning the water into steam. It drives a turbine of appropriate dimensions, generating electricity.

### 3.1. Actuation

**Figure 2:**
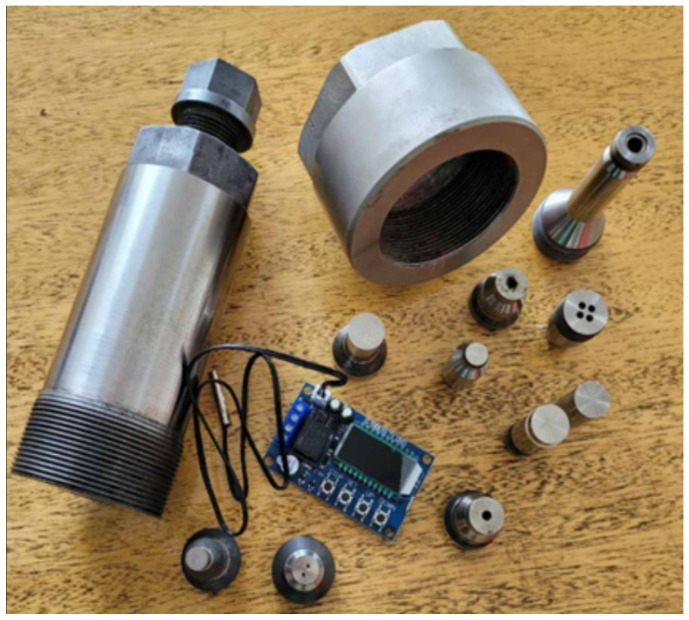
Chamber 1 and some of its components. The ratio of its external diameter d_ext_ to that of channel 2 d_int_ is d_ext_/d_int_ ≥ 4.50. Additionally, pressure F is increased using a multiplier component.

Figure 2 shows the prototype of chamber 1 and some of its accompanying components. The device provides the possibility of creating the monoaxial pressure **F** without a press, by moving piston 3 into the cylindrical channel 2 of chamber 1 by screwing in a corresponding component (“head”) with suitable thread, shown in Figure 2. In this method, the degree of compression for the stages of the chain reaction should be established experimentally in advance. In the setup from Figure 2, specialized measurement equipment is required to study the impact of hydrostatic pressure on the processes of the nuclear reaction. For future nuclear experiments, a neutron moderator is essential to slow down the fast neutrons that are being released. The latter are not effective enough to cause new nuclear fissions with the release of neutrons. In our case, Deuterium D_2_O can be used as a moderator, which converts neutrons into slow-thermal particles. These slow neutrons will more easily maintain the chain reaction. Without a neutron moderator, the nuclear reaction may be ineffective or even stop, and the process is difficult to control. With deuterium the reaction becomes more stable and predictable, especially with hydrostatic pressure [17,19,21,22,23,24,25,26,27].

### 3.2. Sensing Suite

Here, we omit the description of the individual modules of the installation dosimetry sensors, remote data recording, controllers, protective concrete/lead barriers and many other well-known and important systems, devices, equipment, and more, mandatory for the experiments in these cases. Here, we analyze the equipment related to the registration of the new sensor effect by using hydrostatic pressure to control the chain process. The pressure is applied by the modern automated hydraulic press 4, model Bernardo BHP (BHM-Maschine, Germany) with maximum pressure **F** ~ 200 t.

(a) Sika electronic pressure transducer (Lagerwerk GmbH, Germany). A digital pressure transducer with stated accuracy of 0.1% of reading, placed on the hydraulic line of the press. This sensor provides the primary measurement of the force applied to the piston and, via the known piston cross-section, of the hydrostatic pressure transmitted to the enclosed fluid.

(b) Laumas strain-gauge load cell (Laumas Elettronica, Italy). A strain-gauge-based load cell with an integrated digital indicator and a stated accuracy of 0.1% of full scale is used to provide an independent measurement of the load at the piston–chamber interface. The redundant architecture (Sika and Laumas) enables cross-checking of the applied pressure and early detection of load-path anomalies or seal leakage.

(c) Piston displacement channel. The linear displacement of the piston relative to the chamber head, which, combined with the known initial channel volume V_0_, yields the instantaneous fluid volume V(**F**) and therefore the relative volume change ΔV/V_0_(**F**). This channel serves as the bulk sensor of the compressed fluid and is the primary observable in the validation tests reported below.

In a future fissile-material configuration, this suite would be augmented by neutron sensors (a fission chamber or self-powered neutron detector mounted in or near the channel), dosimetry sensors on the chamber exterior, and thermocouples on the chamber wall and cooling circuit. In such a configuration, the Sika and Laumas channels would close the inner actuation loop through the press controller, while the neutron sensors would close an outer reactivity loop used multiplication factor k_eff_(F) experimentally. We note that, in the fissile configuration, D_2_O would additionally serve as a moderator, converting fast fission neutrons into slow (thermal) neutrons and stabilizing the chain reaction [17,19,21,22,23,24,25,26,27].

Additional increase in pressure **F** is achieved using a multiplier made of titanium steel. The pressure is smoothly regulated in increase and decrease modes, as well as maintaining a fixed value of the deformation **F** ≈ const.

### 3.3. Surrogate Fluid and Test Protocol

Before any fissile-material experiment can be undertaken, the mechanical integrity of chamber 1, the performance of the piston seals, and the response of the sensing suite must be characterized in a chemically inert configuration. Chamber 1 was therefore filled with a 50/50 (by volume) mixture of transformer oil and kerosene. This surrogate was selected for its low compressibility at high pressure, its chemical compatibility with titanium and lead, the absence of phase transitions in the pressure and temperature range of interest, and its wide availability. At record-high pressures P, the rate at which the force **F** from the press is applied to the chamber is crucial. A rapid increase in the parameter P poses a real risk of a significant rise in temperature *T*, which is highly undesirable. That is why the correct algorithm for this class of experiments requires a slow increase in pressure P using the press, which we have implemented.

The initial volume of fluid in the chamber at F = 0 was V_0_ = 16,000 mm^3^, and all measurements were performed at room temperature, T = 300 K.

**Figure 3:**
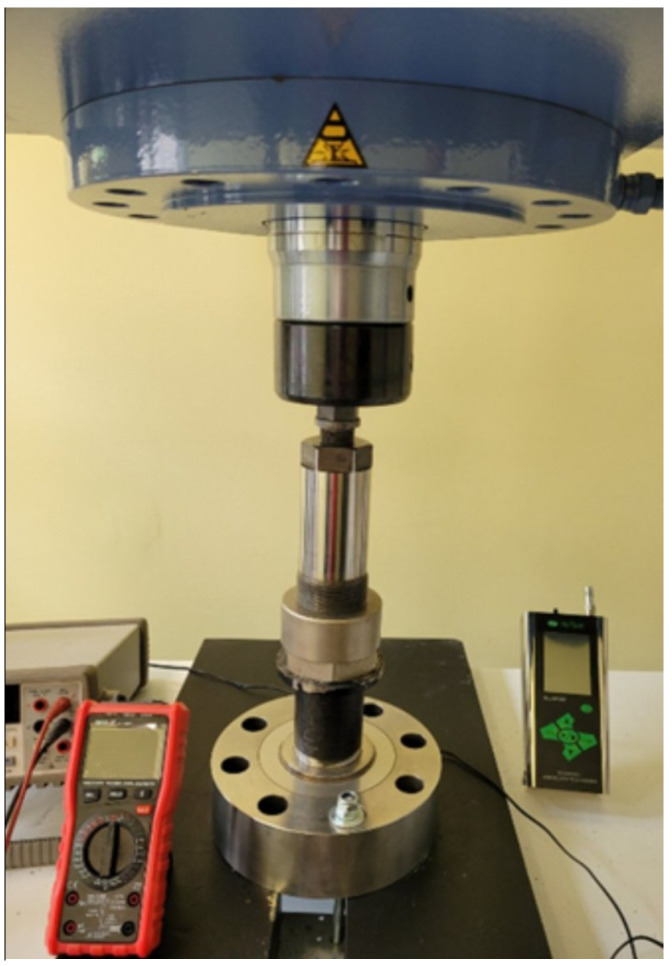
The chamber prototype 1 was tested to about 200 × 10^3^ atm.

Each test consisted of a controlled ramp of the hydraulic press from F = 0 up to a target pressure P not exceeding approximately 2 × 10^5^ atm, followed by a hold phase and a controlled unload. The Sika pressure transducer, the Laumas load cell and the piston 3 displacement were recorded synchronously. The relative volume change ΔV/V_0_ was computed from the piston displacement and the known channel 2 cross-section. Figure 3 shows the chamber prototype under test with the Bernardo press.

**Figure 4:**
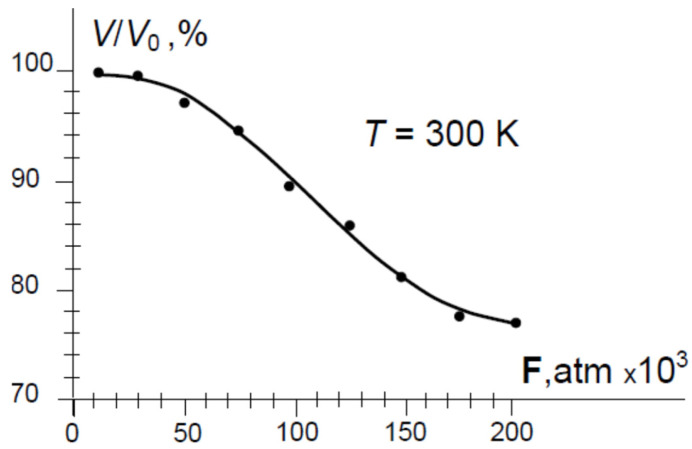
Dependence of the relative change in the volume Δ*V*/*V*_0_ of chamber 1 at hydrostatic pressure **F**. The initial volume of the mixture in chamber 1 is *V*_0_ = 16,000 mm^3^ at room temperature *T* = 300 K.

At this stage, the aim of the experiments is to establish the quality and reliability of the seals of piston 3 with channel 2, which will be used to study the sensor effect in radioactive matter. The change in the volume Δ*V*/*V*_0_(**F**) of the mixture from the parameter **F** > 0 is determined by decreasing the length of the cylindrical piston 3 from the hydrostatic pressure relative to its value at **F** = 0. In these tests, the initial volume *V*_0_ at **F** = 0 of the chamber 1 is 16,000 mm^3^. Figure 4 shows the dependence of the compressibility of the volume Δ*V*/*V*_0_ of the mixture in chamber 1 on the pressure F applied with the press 4, at room temperature *T* = 300 K. The obtained dependence is similar to the behavior of other fluids at high deformation impacts. Experiments have shown that the seals we used on piston 3 under different load modes are suitable for the main purpose of the system.

In the new solution, the neutrons will be retained in the active part of the reactor/chamber 1 as a result of the appropriate design. The functioning of the reactor with slow (thermal) neutrons will be achieved by Uranium 235 or Plutonium 239 in Deuterium, which helps to slow down and control the chain reaction. The mechanical monoaxial pressure of piston 3 in hydrostatic pressure, chamber 1 will be accompanied simultaneously by significant densification of heavy water and convergence of the atoms of the substance. The concentration of _92_U^235^ clusters/atoms in the chamber 1 containing Deuterium should be such that after compaction with high hydrostatic pressure **F** the intensive chain reaction takes place [28,29,30].

## 4. Results

After a certain high-pressure value, **F** >> **F**_0_, the matter should pass from liquid to quasi-metallic phase. In fact, in the fractions of Uranium 235 or Plutonium 239, each neutron captured by the nuclei causes fission with the release of new neutrons. For the first time, it is proposed to control the chain reaction by hydrostatic pressure **F**. This increases the efficiency of catching the amount of neutrons escaping from atoms, accompanied by their absorption by other nuclei, K(**F**) = K_0_(**F**)A(**F**) = 1. The higher the monoaxial pressure exerted by press 4 on piston 3, the greater the increase in the electrostatic interaction between the nuclei, most often repulsion of the charges with one and the same polarity. That is why, with a fixed amount of radioactive material, more neutrons will participate in the chain reaction. By adjusting pressure F in the chamber, the “golden” state of the reactor is reached: K ≈ 1, P ≈ 0. The number of nuclear fissions at fixed pressure **F** ≈ const is practically constant with time t and the nuclear reaction is stable. Apart from densifying the working substance in chamber 1 and increasing the efficiency of the chain process, the new sensor effect reduces the amount of nuclear fuel, which is a strategic raw material. Testing of the described technology and prototype of the device with radioactive matter is forthcoming. Such future tests are necessary to determine the effective neutron multiplication factor **k_eff_** of the proposed device.

The experiments described here were designed to validate the performance of a purpose-built titanium pressure chamber intended for future studies involving Deuterium and Uranium-235. As a preliminary step, weakly compressible inert fluids (a kerosene-transformer oil mixture) were used to characterize the hydrostatic pressure regime of the chamber. At pressures up to 200,000 atm, the mixture exhibits a compressibility of no more than 20% (Figure 4). This surrogate fluid approach allows the pressure envelope to be established safely before introducing radioactive materials. A thorough review of the existing literature was also conducted to identify any functional relationship between high compressive strain and the neutronic behavior of a Deuterium-Uranium-235 fluid mixture. However, all simulations found in the literature are limited to atmospheric pressure conditions, reflecting the absence of primary experimental data on the behavior of fissile materials under high hydrostatic pressure—a gap that underscores the novelty of the sensor phenomenon proposed here. The central open question is by what factor the critical mass is reduced as a function of the compression ratio **F**. Current understanding of the underlying physics suggests that compression of the Deuterium-Uranium system should not diminish the neutron-moderating properties of Deuterium D_2_O, nor reduce its neutron capture cross-section—an assessment consistent with established nuclear physics [30,31,32]. The structural safety of the chamber is ensured by maintaining a wall-thickness-to-channel-diameter ratio of d_ext_/d_int_ ≥ 4.50, which provides an unconventionally large safety margin against pressure-induced failure. Initial test studies conducted with this chamber have been successful (Figure 4). Future experiments will provide the primary dataset necessary for a rigorous theoretical analysis of the compression-driven nuclear processes occurring within the chamber.

## 5. Discussion

One of the advantages of the modular nuclear power plant, Figure 1, Figure 2 and Figure 3, is its simplified design. The many identical pairs of tubes filled with Uranium _92_U^235^ or Plutonium _94_Pu^239^ are eliminated, and there is no high-tech environment slowing down the neutrons spontaneously released in the radioactive material. The process of Uranium fission is initiated by high hydrostatic pressure, which can always be eliminated in experiments with emergency press 4 unloading, where necessary. Also, chamber 1 provides for multiple cycles of loading with uranium _92_U^235^.

In the case of accident or power outages, which are common cases in nuclear energy, the chain reaction will stop. Pressing control through the controllers, one of which is shown in Figure 2, as in robotic technologies, will unload the generated high pressure as quickly as possible, the fission of the radioactive material will pass into state K < 1 and the reactor will fade away. The greatly reduced dimensions of the modular nuclear device and the control of the chain reaction pressure F will enhance the reliability of the reactor. An additional guarantee of safety is that the system is appropriate and can be positioned in a borehole with a diameter of about 0.75 m and depth below the water horizon of soil, about 1500 m, in the faces of abandoned mines or caves. In case of an accident, such locations are easily encapsulated and do not pollute the environment.

In the proposed reactor, the final product, electricity, is produced in the common way. Chamber 1 is placed within a case with high pressure circulating water flow, turning it into steam, which generates electricity through a turbine [1,2,3]. As with all nuclear power plants, the materials used to build the reactor must be of exceptional quality, as they operate at high temperatures and in a field of neutrons, alpha, beta and gamma rays, and fission fractions. The titanium (or beryllium steel) proposed in the design, on the one hand, retains the neutrons, and on the other, is an extremely strong metal composite. Moreover, it is radiation-resistant/proof. There are many modifications of presses available, but the main requirement is to generate sufficient pressure **F** in the experiments to initiate the chain reaction.

## 6. Conclusions

The novelty of the proposed sensor effect is that high hydrostatic pressure serves as the mechanism by which a chain reaction is initiated and sustained. An essential feature of the described phenomenon is that, with the help of hydrostatic pressure and at smaller amounts of G than the critical mass of Uranium, G < G_0_, it is possible to achieve conditions for a chain reaction to take place. This process is easily controlled and features increased safety. In addition to the many engineering advantages, the result is environmentally friendly, as the dangers of nuclear pollution are significantly reduced. Small modular reactors, similar to the one presented above, are cheaper, and the time for installation, construction and legalization is shortened. So far, the chamber, its operation at high pressures and the controllers have been experimentally tested. The content of the reactor core area can be replaced by Uranium in powdery state. When subjected to high uniaxial deformations in the chamber, the atoms of the metal in such a state also come closer, affecting the chain process. The overall testing of the effect and technology may be carried out under appropriate control. The new modular devices can be used in autonomous robots, air and underwater drones, urban heating, and more.

## Data Availability

The data supporting the findings of this study are available within this article. Further inquiries can be directed to the corresponding author.
